# New insights into T cell metabolism in liver cancer: from mechanism to therapy

**DOI:** 10.1038/s41420-025-02397-w

**Published:** 2025-03-23

**Authors:** Jie Xiao, Ting Liu, Fanxin Zeng, Jinhua Zhang

**Affiliations:** 1 State Key Laboratory of Targeting Oncology, National Center for International Research of Bio-targeting Theranostics, Guangxi Key Laboratory of Bio-targeting Theranostics, Collaborative Innovation Center for Targeting Tumor Diagnosis and Therapy, Guangxi Talent Highland of Major New Drugs Innovation and Development, Guangxi Medical University, Nanning, China; 2 College of Life Science and Bioengineering, Beijing Jiaotong University, Beijing, China; 3 School of Life Science and Technology, Jinan University, Guangzhou, Guangdong China; 4 Department of Clinical Research Center, Dazhou Central Hospital, Dazhou, Sichua China

**Keywords:** Cancer metabolism, Immunization

## Abstract

Liver cancer is the sixth most common cancer worldwide and the third most common cause of cancer mortality. The development and progression of liver cancer and metastases is a multifaceted process involving numerous metabolic pathways. T cells have a protective role in the defense against cancer, and manipulating metabolic pathways in T cells can alter their antitumor activity. Furthermore, Liver cancer and T cell nutrition competition lead to T cell dysfunction through various molecular mechanisms. Some nanomaterials and drugs can improve T cell metabolism and promote the anti-liver cancer function of T cells. This review discusses the current literature regarding metabolic changes in liver cancer, the role of T cells in liver cancer, T cell metabolism in liver cancer, and targeted T cell metabolism therapy for liver cancer. The promise and challenges of studying target T cell metabolism for treating liver cancer are also addressed. Targeting T cell metabolism is a promising approach for treating liver cancer.

## Facts

- The function of T cells is closely linked to metabolism. Tumor and T cell metabolism have similar patterns and characteristics.
- The metabolism of T cells plays a crucial role in antitumor immunity.
- Regulating T cell metabolism can improve liver cancer sensitivity to immunotherapy and chemotherapy.
- The unique metabolic characteristic of T cells in liver cancer is that altered T cell metabolism plays different roles in Nonalcoholic Steatohepatitis (NASH), Autoimmune hepatitis (AIH), and Hepatocellular carcinoma (HCC) development.

## Open Questions

- How should we investigate the molecular pathways underlying T cell metabolism in liver cancer?
- Can T cells enhance the inflammatory response in NASH through metabolism?
- Can balancing T cell metabolism contribute to alleviating liver cancer?
- which treatments can enhance the killing ability of T cells while reducing the growth of liver cancer cells?

## Introduction

Liver cancer is a relatively common type of cancer that is a significant cause of death worldwide. It is the sixth most prevalent cancer type and the third leading cause of cancer-related mortality [1]. Liver cancer is more common in poorer countries considering the high incidence of hepatitis B and C infections, which are the primary causes of the disease. Nonalcoholic fatty liver disease (NAFLD), viral hepatitis, and alcohol consumption are risk factors for hepatocellular carcinoma (HCC), which accounts for approximately 90% of all liver cancers [2–4]. Despite significant progress in treatment, liver cancer remains a considerable challenge, and more research is needed to develop effective therapies that can improve the prognosis for liver cancer patients.

Glucose, amino acids, lipids, and mitochondrial function primarily drive the metabolism of liver cancer. For example, the decreased expression of genes associated with aerobic metabolism and glycolysis in HCC cells can inhibit liver cancer growth [2]. Moreover, HCC cells can produce lipids de novo while simultaneously increasing the absorption of fatty acids (FAs) [4]. A better understanding of the complex metabolic pathways within HCC may lead to the development of novel therapeutics. To promote their growth, tumor cells require significant nutrition to generate metabolites that contribute to modifying the tumor microenvironment (TME) [5, 6]. These changes in the TME alter immune cell metabolism and anticancer immunity, thus enabling tumor cells to avoid immune surveillance and grow abnormally [7].

T cells play an important function in the body’s immune response toward liver cancer by detecting and killing malignant cells [8, 9]. T cells have a protective role in the defense against cancer. T cells produce numerous cytokines, such as TNF-α, IFN-γ, and GzmB, which induce cancer cell death [10]. Patients with substantial CD8^+^ T cell infiltration experience a markedly improved survival time compared with those with minimal infiltration [11]. CD4^+^ T cells mediate antitumor immunity through direct cytotoxicity [12]. Following the activation of naive T lymphocytes, their metabolism is reprogrammed to generate ATP for the development, proliferation, and synthesis of effector molecules [13]. Cell signaling pathways and epigenetics are regulated by metabolic activity, significantly affecting T cell development and fate [14]. Furthermore, T cell activation stimulates metabolism, promoting the transition from inactivity to growth and proliferation [15]. Cancer cell metabolism can limit nutrients and accumulate waste. In addition to producing inhibitory ligands that directly affect T cell function, the restriction of T cell metabolism may reduce effector T cell function, while augmenting suppressive regulatory T cells, which abolishes the effects of immunotherapy [16].

In the TME, T cells compete with liver cancer cells for glucose and lipids. They are exposed to lactate and other metabolic products dispersed throughout the environment. Lactate can affect immunological tolerance in the TME by attracting regulatory T cells (Tregs) [17]. This milieu causes substantial metabolic and functional alterations in tumor-infiltrating T cells, facilitating the escape of liver cancer cells from immune surveillance [18]. In contrast, liver cancer cells outcompete T cells for nutrients required to support their growth, resulting in T cell dysfunction and HCC malignant progression [19]. This review offers a comprehensive summary of recent advancements in T cell metabolism in the context of liver cancer. It also proposes novel therapeutic approaches for targeting T cell metabolism in treating liver cancer.

## General characteristics of liver cancer metabolism

**Fig. 1:**
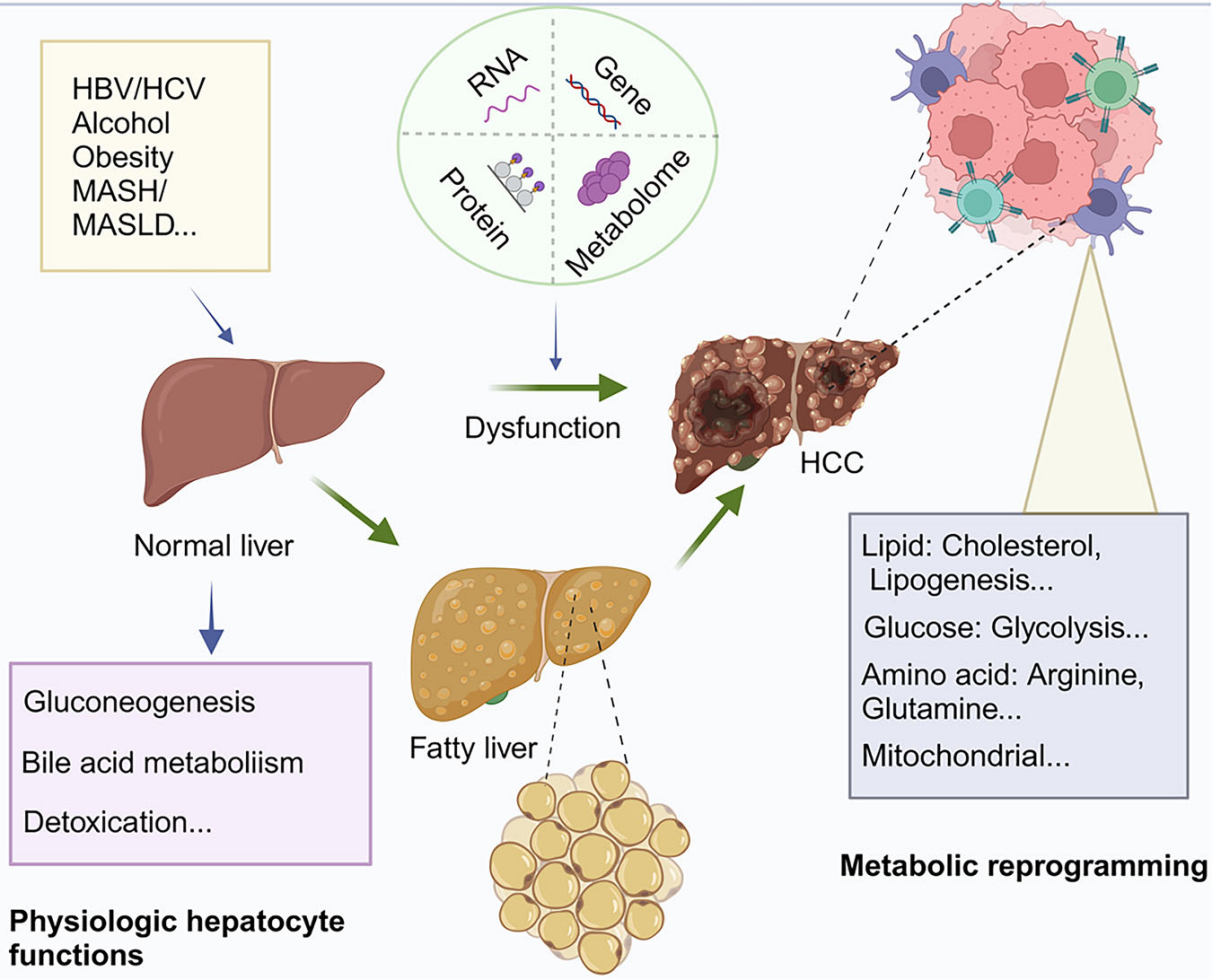
Metabolic alterations in hepatocellular carcinoma (HCC). The induction and metabolic modifications of liver cancer. HBV hepatitis B virus, HCV hepatitis C virus, MASH metabolic dysfunction-associated steatohepatitis, MASLD metabolic dysfunction-associated steatotic liver disease.

Metabolic changes accompany the liver carcinogenesis. During the progression of liver cancer, gluconeogenesis, detoxication, bile acid metabolism, and other typical hepatocyte metabolic functions are decreased, and these metabolic changes are accompanied by an increase in tumor progression [20, 21]. In addition, liver cancer cells require much energy for growth. Therefore, it is essential to understand the basic metabolic processes responsible for their development in order to develop effective treatments. Several studies have examined the metabolic patterns and processes associated with liver cancer metabolism (Fig. 1 and Table 1).

**Table 1:** Overview of the metabolic molecular mechanisms of liver cancer.

Gene symbol	Target gene	Metabolic type	Study result	References
Nrf1α	PI3K-AKT-mTOR	Glycolysis	Inhibits glycolysis in the HCC	[25]
RFX6	PGAM1	Glycolysis	Promotes glycolysis in the HCC	[28]
FTO-IT1	GLUT1, PKM2, c-Myc	Glycolysis	Promoting glycolysis in the HCC	[27]
ONECUT3	PDK1	Glycolysis	Promotes glycolysis in the PDAC	[54]
HNF4α		Amino acid metabolism	Promotes sulfur amino acid metabolism in liver cancer	[32]
SLC25A15	SLC1A5	Amino acid metabolism	Inhibit glutamine metabolism in the HCC	[34]
OGDHL		Amino acid metabolism	Inhibit glutamine metabolism in the HCC	[37]
TM4SF5	FKBP8, NPC1	Lipid metabolism	Promoted mitophagy and cholesterol transport to mitochondria	[40]
RBM45	ACSL1, ACSL4, Rictor	Lipid metabolism	Promotes de novo lipogenesis in HCC	[41]
CircLARP1B	HNRNPD	Lipid metabolism	promoting fatty acid synthesis in HCC	[43]
FTCD	PTEN/Akt/mTOR, SREBP2, PPARγ	Lipid metabolism	Inhibit lipid accumulation and hepatocarcinogenesis	[44]

### Glucose metabolism in liver cancer

Adenosine triphosphate (ATP) production in tumor cells results from two opposing metabolic mechanisms: glycolysis and oxidative phosphorylation (OXPHOS). Both pathways generate energy, helping sustain metabolic homeostasis [22]. During glycolysis, glucose is broken down into pyruvate, oxidized, and delivered to the mitochondria, transforming it into more ATP. Other fuel sources, including FA and amino acids, contribute to the OXPHOS process. The OXPHOS mechanism is primarily responsible for creating ATP, the primary fuel source for cells [23]. It is well-known that glycolysis plays an important role in HCC development. Aerobic glycolysis can change the TME of HCC by promoting angiogenesis, local invasion, and possibly immune evasion [24].

The PI3K/AKT signal pathway is frequently activated in HCC and plays an important role in tumor formation [2]. Furthermore, activating the PI3K-AKT-mTOR signaling network induces the expression of glucose- and de novo lipid synthesis-related genes [25]. Loss of KDM6A in mice suppresses the PI3K-AKT-mTOR signaling pathway, leading to reprogramming into glucose and lipid metabolism in HCC [26]. Overexpression of GLUT1 (glucose transporter 1) and PKM2 (pyruvate kinase isozyme type M2) increases glycolysis in HCC [27]. RFX6 expression is increased in HCC tissues, which upregulates the expression of phosphoglycerate mutase 1 (PGAM1). The development of HCC is further promoted by enhanced glycolysis resulting from elevated PGAM1 protein levels [28]. Although the significance of glucose metabolism in liver cancer cells is well-documented, other metabolic pathways also provide energy during liver cancer development [24].

### Amino acid metabolism in liver cancer

Tumor cells rely on amino acid absorption to maintain key processes. Methionine constitutes one of several essential dietary amino acids [29]. Because methionine availability affects cellular metabolism and proliferation, the cell cycle is irreversibly arrested when liver cancer cells are depleted of methionine. Methionine catabolism induces cell cycle arrest and DNA damage in liver tumors, which results in cell death [30]. Additionally, Li et al. discovered that limiting methionine to human liver tumor cells causes irreversible cell cycle arrest. Reducing methionine adenosyl transferase 2A (MAT2A) promotes cell cycle arrest and DNA damage in liver cancer cells, leading to cell senescence [31]. Furthermore, combining methionine depletion with sorafenib therapy in liver tumors represents a potential treatment approach. HNF4α is a regulator of hepatic sulfur amino acid metabolism. Knocking down HNF4α in liver cancer cells attenuates resistance to methionine restriction and sorafenib, accelerates the epithelial-mesenchymal transition, and promotes cell migration [32].

Glutamine is a common substrate for many amino acid transporters. Glutamine bioavailability in cancer can be regulated by amino acid transporters [33]. Deficiency in solute carrier family 25 member 15 (SLC25A15) causes reprogramming of glutamine metabolism, promoting HCC development [34]. Branched-chain amino acid (BCAA) catabolism is triggered in liver cancer cells that lack glutamine [35]. A multi-omics strategy to analyze liver carcinogenesis and regeneration revealed that a gradual decrease in BCAA metabolism promotes tumor development and proliferation [36]. Oxoglutarate dehydrogenase-like (OGDHL) is a rate-limiting component of the main mitochondria multi-enzyme OGDH complex (OGDHC). Silencing OGDHL increases HCC formation and survival by modifying glutamine metabolic pathways [37].

### Lipid metabolism in liver cancer

Lipid metabolic reprogramming plays an important role in hepatocarcinogenesis. Excessive lipid accumulation promotes obesity and increases the risk of HCC [38]. Biologically, a high-fat diet may boost AKT kinase activity, thereby causing nonalcoholic steatohepatitis (NASH) and liver cancer [39]. More molecular mechanisms of lipid metabolism in liver cancer need to be explored. In mouse models, mitophagy and cholesterol transport to the mitochondria positively correlate with liver cancer [40]. RBM45 is differentially expressed in HCC and affects how HCC cells behave and manage fats. In HCC, RBM45 directly targets two important enzymes involved in synthesizing long-chain FAs, namely, ACSL1 and ACSL4. This results in new fats in HCC cells [41]. Depletion of Trim26 in hepatocytes alters liver metabolic equilibrium, leading to glucose metabolic dysfunction, lipid buildup, hepatic inflammation, and fibrosis, all of which accelerate the progression of the NASH phenotype [42]. In an induced HCC model, CircLARP1B-deficient animals exhibit decreased metastasis and lipid buildup; On the other hand, CircLARP1B increases fatty acid production, thus enhancing cellular metastasis and lipid buildup in HCC [43]. Loss of formimidoyltransferase cyclodeaminase (FTCD) stimulates the PPARγ and SREBP2 signaling pathways, resulting in lipid buildup and hepatocarcinogenesis [44].

## T cell metabolism in the TME

**Fig. 2:**
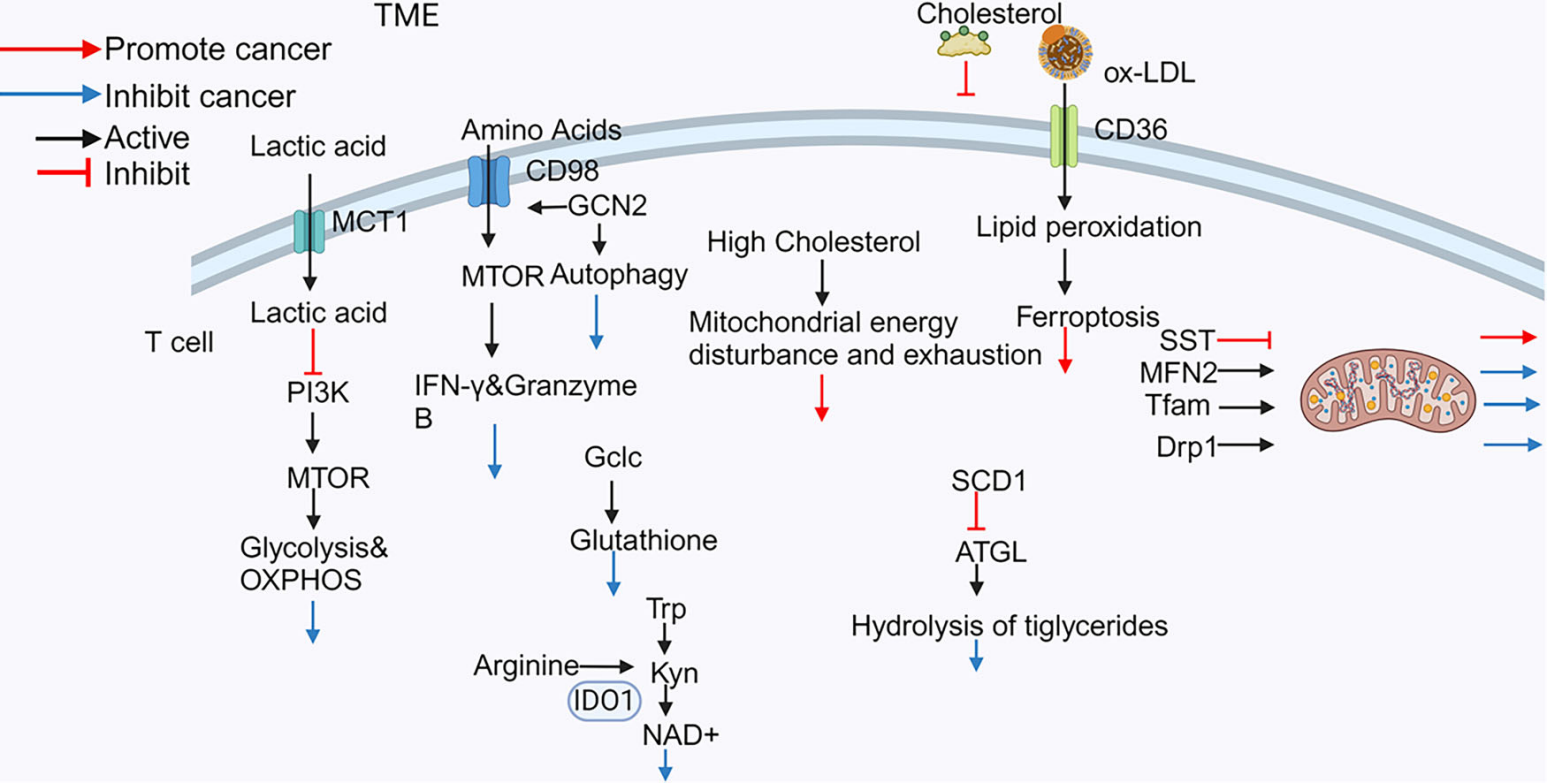
Metabolic patterns of T cells in the tumor microenvironment (TME). T cell metabolism is crucial for its function, and T cells obtain nutrients through various mechanisms to maintain their stability. MCT1 monocarboxylate transporter 1, Kyn kynurenine, NAD nicotinamide adenine dinucleotide, Trp tryptophane; SIRT1 Sirtuin 1, GCN2 general control nonderepressible 2, PD-1 programmed death protein 1; GLUT1 glucose transporter 1; IDO1: Indoleamine 2,3-dioxygenase 1.

The function of T cells is closely linked to metabolism. Tumor and T cell metabolism have similar patterns and characteristics (Fig. 2).

### Glucose metabolism affects the antitumor effect of T cells

T cell activation and differentiation rely on glucose metabolism, which is required to sustain an antitumor T cell response [45]. T cells require metabolic reprogramming during activation, resulting in various functional fates [46]. Quiescent T cells generate energy through OXPHOS, whereas activated T cells utilize glycolysis. T cells increasingly rely on glycolysis rather than OXPHOS as their effector activities develop [47]. Various types of T cells generate energy through glucose metabolism. For example, CD4^+^ T cells rely on glycolysis and Tregs require glucose for proliferation. However, poor glycolysis inhibits CD4^+^ T cell differentiation [48, 49]. Tregs exhibit higher PD-1 expression than effector T cells in highly glycolytic tumors [50]. Tregs can use some metabolites detrimental to effector T cells, such as lactic acid, which is a toxic metabolite to functional T cells but can support Treg growth and function [51]. Additionally, naive T cells produce energy and signaling chemicals through glycolysis, mitochondrial metabolism, and OXPHOS [52].

Tumor cells require a substantial amount of glucose for glycolysis, resulting in a glucose deficiency in the TME. Conversely, glucose deprivation in the TME inhibits T cell function and activity [53]. Glycolytic metabolism is inversely associated with tumor T cell infiltration [54]. Tumor glycolysis creates enormous amounts of lactate, which suppresses the immune response in glycolytic cancers. Intratumoral lactate buildup and acidosis decrease T cell activity and tumor immunity [55]. Cancer cells compete with tumor-infiltrating T lymphocytes (TILs) for glucose uptake, which results in a glucose-deficient TME with significant lactate buildup. This inhibits CD8^+^ TIL effector activity while promoting the immunological suppression of Tregs [56]. Disrupted glucose metabolism in CD8^+^ T cells occurs through inhibition of the PI3K/AKT/mTOR signaling pathway, which results in decreased cytokine production [57]. Glycolytic enzymes play an important role in tumor nutrition competition and are expressed at high levels in many cancer types. They are also directly related to survival [58, 59]. Moreover, glycolytic enzyme inhibitors enhance memory T cell formation and tumor clearance [60]. A deficiency in glycolytic enzymes, such as Glut1 and Gpi1 (glucose-6-phosphate isomerase 1) in tumor cells, can enhance a T cell-mediated antitumor response [61]. Furthermore, Lactic acid, a toxic metabolite to activating T cells, supports Treg growth and function [51], whereas lactate catabolism decreases Treg induction, promotes antitumor immunity, and inhibits tumor growth in mice [62]. Aberrant glucose metabolism is a characteristic of cancer and the TME produces metabolic challenges to T cells, thus impairing the antitumor immune response [45]. Therefore, targeting glucose metabolism offers a promising approach to enhancing T cell function in the TME.

### Amino acid metabolism affects the antitumor effect of T cells

Amino acids are essential biomolecules for protein synthesis and their metabolic pathways play an important role in T cell activity. Tryptophan is required for T cell function because its metabolism controls the immune system by serving as an immunomodulator [63]. However, insufficient tryptophan levels in the TME can limit effector T cell activation, while increasing the number of Tregs [51]. Renal cell carcinomas cells can increase tryptophan metabolism by upregulating indoleamine 2,3-dioxygenase 1 (IDO1), which results in T cell dysfunction and Tregs infiltration. Conversely, reduced IDO1 expression ameliorates cytotoxic T cell inhibition and enhances the response to anti-programmed cell death protein 1 (anti-PD-1) therapy [64]. Overexpressing IDO1 in a mouse model of prostate cancer increased the number of CD8^+^ T cells and decreased natural killer T cells. IDO inhibitors can activate CD8^+^ T lymphocytes by increasing tryptophan levels and reducing PD-1 expression [65–67]. Activating autophagy and the CD98-mTOR axis with the GCN2 agonist halofuginone can improve the amino acid-deficiency response in T cells and antitumor efficacy [68]. Furthermore, glutarate, which is a byproduct of amino acid metabolism, can affect CD8^+^ T cell development and enhance tumor cell cytotoxicity [69].

Other amino acids regulate T cell activity in malignancies. For example, lysine is a biosynthetic, antioxidant, and energy source. A lysine-restricted diet combined with anti-PD-1 therapy slows tumor growth [70]. Dietary supplementation containing methionine boosts antitumor immunity and reduces tumor development. Cysteine depletion in T lymphocytes inhibits glutathione synthesis and promotes CD36-mediated lipid absorption. Overexpression of the glutamate-cysteine ligase catalytic subunit (Gclc) enhances glutathione production, inhibits CD36 upregulation, and boosts T cell antitumor immunity [71]. Thus, it is important to understand the physiological requirement for amino acids that modulate T cell activity.

### Lipid metabolism affects the antitumor effect of T cells

Normal T cell immunity relies on the progression of lipid metabolism. Cholesterol and FAs present in the TME can contribute to CD8^+^ TIL malfunction [72]. During T cell priming and activation, cholesterol can disrupt the structure of the T cell receptor, decreasing its ability to detect and respond to threats [73]. Cholesterol is an important component of lipid metabolism. High cholesterol levels promote interactions between the endoplasmic reticulum (ER) and mitochondria in CD8^+^ T cells, causing CD8^+^ T cell disruption and exhaustion of mitochondrial activity in colorectal cancer [74]. Furthermore, certain lipids can enhance the function of CD8^+^ T cells. For instance, Linoleic acid (LA) therapy improves communication between the ER and mitochondria. Exposing CD8^+^ T cells with LA can enhance their antitumor activity. Conversely, certain fats can damage the mitochondria in CD8^+^ T cells, leading to a reduced antitumor response [75]. Regarding T cell priming and activation, cholesterol disrupts the structure of the T cell receptor, reducing immunodetection; however, cholesterol enhances T cell receptor clustering and signal transmission [73].

ACLY is important for the formation of cholesterol and FAs. Xiang W et al. demonstrated that ACLY inhibition in immunocompetent mice increases PD-L1 immune checkpoint expression in cancer cells, which results in T cell malfunction, immunosuppression, and a compromised antitumor effect [76]. Additionally, Stearoyl-CoA desaturase-1 (SCD1) is involved in the depletion of FAs. The absence of SCD1 in T cells results in the hydrolysis of triglycerides and phosphatidylcholine by adipose triglyceride lipase. Moreover, SCD1 acts as an endogenous brake on Treg development and exacerbates the likelihood of autoimmunity [77]. These findings suggest that cholesterol and FAs are negative factors for effector cells, for example, CD8^+^ T cells in the TME, whereas altering lipid metabolism can enhance their antitumor activity.

### Mitochondrial metabolism affects the antitumor effect of T cells

Mitochondrial dynamics and metabolic pathways regulate T cell fate [78]. Mitochondria perform a range of cellular tasks, including energy and ROS production. They also play a role in signaling metabolites, modulating cell signaling, and apoptosis. Mitochondria play a role in cancer by sensing mitochondrial stress, which allows cells to adapt to environmental changes [79]. Significant changes in the mitochondria occur within the first 24 h following T cell activation. This imparts mitochondria with a distinct metabolic style. Purine and thymidine are synthesized through single-carbon metabolism and salvage mechanisms fueled by serine, promoting T cell growth and survival [15, 80]. Mitofusin-2 (MFN2) knockdown in CD8^+^ T cells suppresses mitochondrial metabolism and function, thus accelerating tumor growth [81]. The inhibition of effector CD8^+^ T cell growth and clonal proliferation is caused by deleting the protein tyrosine phosphatase mitochondrial 1 (PTPMT1). A shift in mitochondrial substrates and metabolic inflexibility causes DNA mutations, oxidative stress, and apoptosis in PTPMT1 knockout cells. PTPMT1 knockout impairs the mitochondrial metabolism of CD8^+^ T cells and promotes melanoma growth by increasing CD8^+^ T cell fatigue and malfunction [82]. Somatostatin (SST) inhibits T cell proliferation by affecting T cell mitochondrial respiration [83]. Mitochondrial transcription factor A (Tfam) is necessary for mitochondrial respiration. Following the deletion of Tfam in Tregs, Treg stability and tumor rejection are affected [84]. Dynamin-related protein 1 (DRP1)-mediated mitochondrial fission is important for T cell activation, proliferation, and migration. High levels of Drp1 affect T cell activation by enhancing the T cell-induced suppression of lung cancer cells, which increases CD8^+^ T cell infiltration into the tumor and amplifies the immune system response to treatment with the PD-1 inhibitor pembrolizumab [85]. Regulating T cell mitochondrial metabolism significantly affects T cell antitumor activity.

### T cell function and role in liver cancer

The immune system is an important defensive mechanism against malignancy and tumor progression. However, cancer cells have developed various mechanisms for T cell depletion, which is an adverse factor in HCC, circumventing immune monitoring, such as promoting T cell fatigue blocking T cell penetration, and inducing immunosuppressive factors [86]. Modulating the proliferation and activation of T cells and increasing the ratio of CD8^+^ T cells in peripheral blood improve the therapeutic efficiency of antitumor immunotherapy [87]. TILs and chimeric antigen receptor (CAR) T cells have shown extraordinary efficacy in treating relapsed/refractory tumors [88]. Furthermore, T cells play different functions in the development of liver cancer. T cells are identified as a major immunological component in the MASH-induced HCC transition [89]. CD8^+^ and CD4^+^ T cells drive fibrosis and. inflammation in NASH. CD8^+^ T cells use myeloid cell MHC class I expression to promote inflammation and fibrosis in NASH. Furthermore, CD8^+^ T cells play a minor role in disease progression under non-obese conditions in NASH [90, 91]. IFN-γ plays a crucial role in regulating perforin control of NASH development. Increased intrahepatic activation and proliferation of CD4^+^ T cells in NASH, along with increased IFN-γ and TNF-α production, activation of the pro-inflammatory response in NASH [92, 93]. In the transition from HBV infection to cirrhosis and eventual development of HCC, damage to CD8^+^ T cell effector function and differentiation [94]. In mild to severe NASH and early fibrosis, hepatic NKT cells are reduced, resulting in a pro-inflammatory state. In severe NASH and advanced liver fibrosis, NKT cells have a role in suppressing the NASH-related fibrogenic response [95]. Effector T cells play an important function in the immune response to cancer. The depletion of CD8^+^ T cells is detrimental to HCC [96]. Recent studies have shown that modulating the killing function of CD8^+^ T cells markedly enhances anti-HCC immunity. Immunity-related GTPase M (IRGM) is a novel regulator of PD-L1 that inhibits CD8^+^ CTL infiltration and function in HCC, resulting in cancer progression [97]. GLI-similar 1 (GLIS1) downregulation in CD8^+^ T cells slows cancer progression, increases infiltration, and enhances the anti-PD1 response in HCC. CD8^+^ T cells are essential in eradicating acute hepatitis B virus (HBV) infection and liver damage [98]. However, HBV-specific CD8^+^ T cells can lead to HBV-associated HCC. In HBV progressive chronic inflammation, HBsAg-specific CD8^+^ T cells are created, and they selectively and persistently trigger hepatocyte death with growing chronic inflammation. HBsAg-specific CD8^+^ T cell depletion or deficiency prevents HCC [99]. Moreover, Treg cells play a role in promoting the development of liver cancer. The Treg subpopulation is selectively elevated in NASH. Depleting Tregs significantly reduces HCC development and progression in NASH [100]. Treg cell-derived AREG is stimulated to promote liver fibrosis by epidermal growth factor receptor (EGFR) activation [101]. Tregs also enhance the polarization of M2 macrophages, promoting HCC development, migration, and invasion [102]. Tregs perform several functions in the progression to HCC. Tregs play a protective role in limiting chronic inflammation during liver fibrosis, but in the liver TME, an increase in Tregs’ numbers can be deleterious [103].

## T cell metabolism in liver cancer

**Fig. 3:**
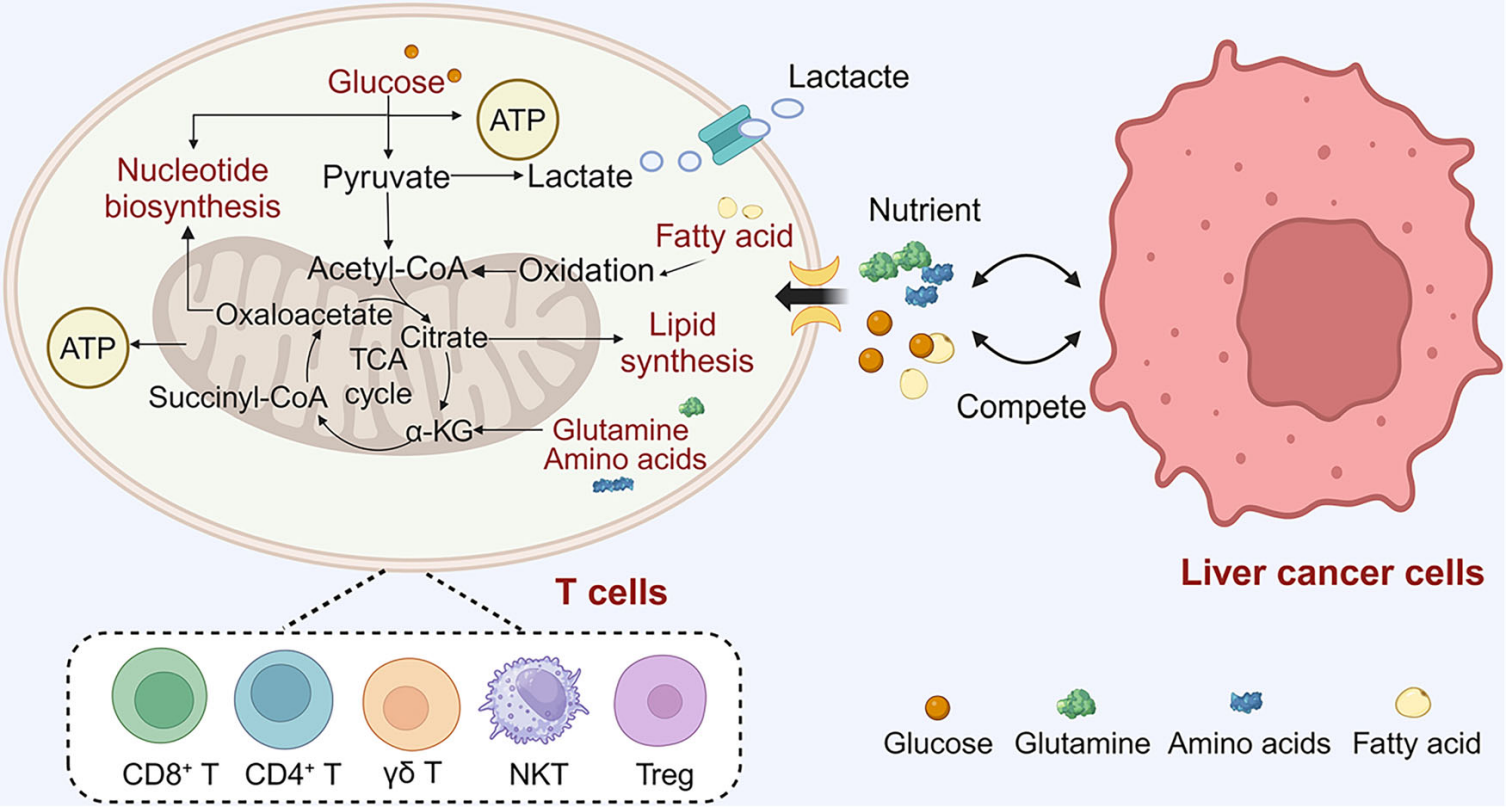
The nutritional competition between liver cells and T cells in TME. The competition-caused deficiency of glucose, amino acids, fatty acids, glutamine, and other nutrients affects the function of T cells, including CD8^+^ T cells, CD4^+^ T cells, γδ T cells, NK T cells, Treg, and so on. α-KG α-Ketoglutarate, ATP adenosine triphosphate, TCA trichloroacetic acid.

**Fig. 4:**
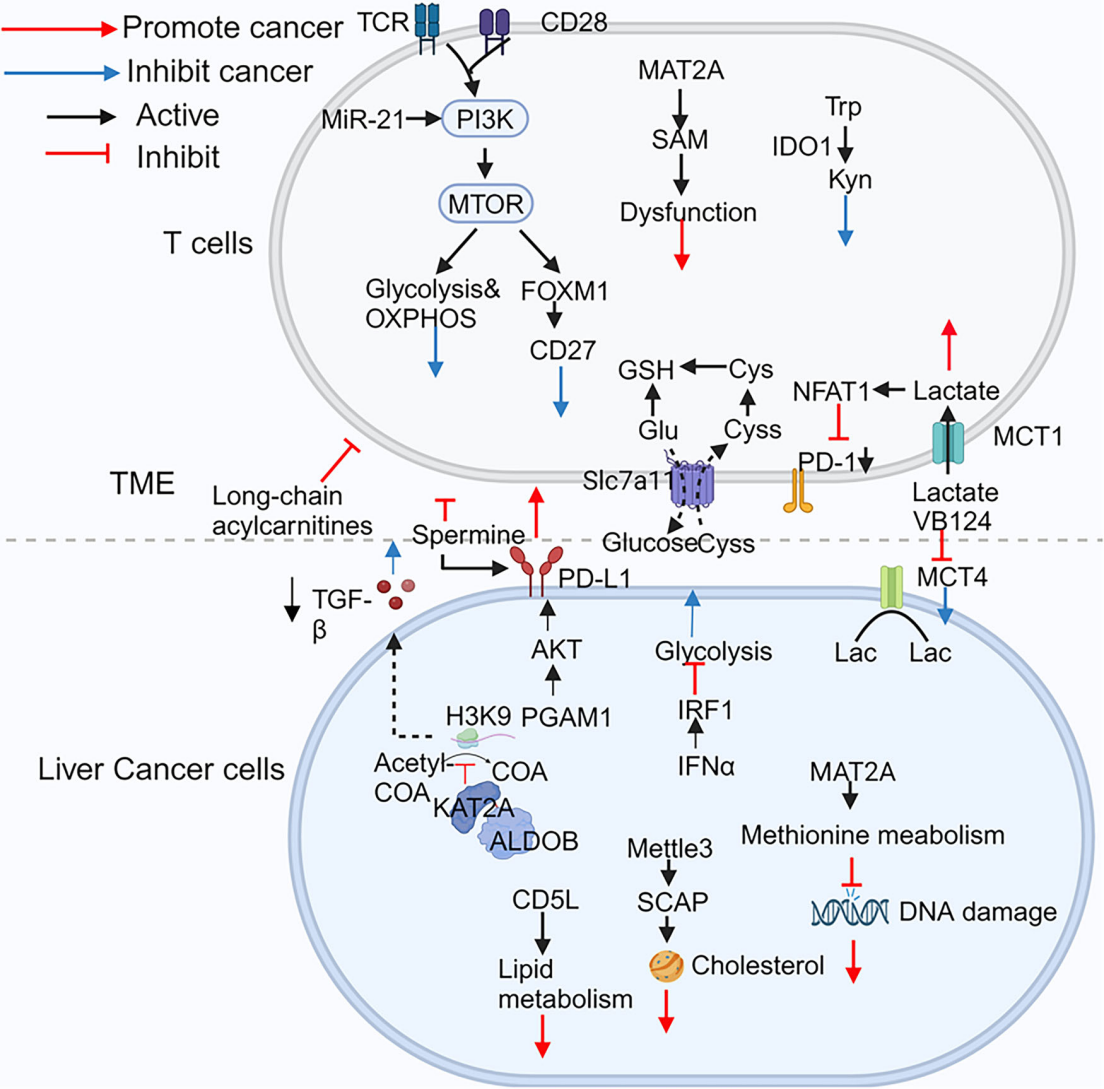
The molecular mechanism of nutrition competition between liver cancer cells and T cells. Liver cancer cells in the TME compete with T cells for glucose, amino acids, and lipids, affecting T cell metabolism through different pathways. ALDOB fructose-1,6-bisphosphate aldolase B, CD5L CD5 molecular-like, CySS cysteine, Cys cysteine; Glu glutamine, Lac lactic acid, GSH glutathione, IFNα interferon alpha, Kyn kynurenine, MCT4 monocarboxylate transporter 4, MCT1 mnocarboxylate transporter 1, MAT2A methionine adenosyltransferase 2, Pyr pyruvate, SAM S-adenosylmethionine, SLC7A11 solute carrier family 7a member 11, Trp tryptophane; Tregs regulatory T cells.

Liver cancer is a common type of cancer that remains a global health concern. Studying the metabolic competition and molecular mechanisms between liver cancer and T cells can offer valuable insight and improve the treatment and prognosis of liver cancer patients (Fig. 3). The discussion below summarizes the recent literature regarding the metabolism and molecular processes associated with T cells in HCC (Fig. 4).

### The role of glucose metabolism between T cells and liver cancer cells

**Fig. 5:**
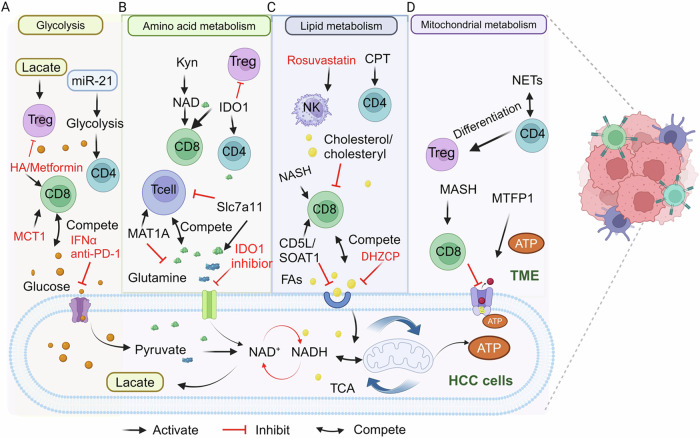
Immune regulation by nutrient competition and potential metabolic inhibitor. **A** Equilibrium glycolytic reaction. **B** Amino acid balance. **C** Lipid metabolism competition. **D** Mitochondrial metabolism between HCC cells and T cells. CPT carnitine palmitoyltransferase, FAs fatty acids, HA Hyaluronic acid, IFNα interferon alpha, Kyn kynurenine; hyaluronan, MCT1 monocarboxylate transporter 1, MAT1A methionine adenosyltransferase 1, MTFP1 mitochondrial fission process 1 protein, NETs neutrophil extracellular trap.

The level of glycolysis activity in tumor cells influences the function of T lymphocytes. The regulation of glycolysis in tumor cells enhances glucose concentration in the TME and improves T cell metabolism. In HCC cells, increased glucose uptake promotes their proliferation. Similarly, enhanced glucose uptake by CD8^+^ T cells supports the effector function of CD8^+^ T cells. The metabolism in the TME is a complex process, and liver cancer’s metabolic activity also impacts T cells’ metabolism. Interferon alpha (IFNα) alters glucose metabolism in the TME in HCC. IFNα inhibits HIF1α signaling by reducing FosB transcription in HCC cells, creating a high-glucose microenvironment. This could improve the nutrient deficiency in effector T cells. Combining IFNα with anti-PD-1 therapy decreases glycolysis and glucose uptake in HCC cells and increases glycolysis in TILs [104]. PGAM1 is considered a novel immunometabolic target. Inhibition of PGAM1 leads to HCC cell ferroptosis by decreasing Lipocalin (LCN2) and inducing energy stress. This inhibition also increases anticancer effects by enhancing CD8^+^ T cell infiltration [105]. The intense competition of glucose between liver cancer cells and CD8^+^ T cells results in the exhaustion of T cells due to the overexpressed key enzymes of liver cancer cells that control glycolysis, giving tumor cells an advantage in glucose uptake. Fructose-1,6-bisphosphate aldolase B (ALDOB) in HCC has been shown to organize metabolic programming that promotes HCC. Furthermore, ALDOB negatively correlates with CD8^+^ T cells and enhances the number of Tregs infiltrating human HCC tumors [106]. Moreover, the high expression of PKM2 is found in patients with HCC, and PKM2 is associated with poor outcomes and immunosuppressive CD8^+^ T cells [107]. Decreasing glucose metabolism in Th17 cells may alleviate inflammation severity in NAFLD. Deleting PKM2 in Th17 cells significantly alleviates ihTh17-centric inflammation and NAFLD severity [108]. Metformin is a medication used to treat type 2 diabetes mellitus. Recent studies suggest that it may also inhibit the onset and progression of liver cancer. The glucose uptake capacity and mitochondrial function in CD8^+^ T cells are gradually diminished in NASH progression. Metformin treatment for NASH can enhance the metabolic function of CD8^+^ T cells and improve the effectiveness of PD-1 therapy [109]. The increased glucose metabolism in CD4^+^ T cells suppresses liver cancer cell proliferation. In contrast, the heightened glucose metabolism in CD4^+^ T cells may contribute to developing and progressing NAFLD, liver fibrosis, and autoimmune hepatitis (AIH). NAFLD is linked to the accumulation of CD4^+^ T cells in the liver, which have a pathogenic character, express CXCR3, and produce pro-inflammatory cytokines. Inflammatory hepatic CD4^+^ T cells have a specific metabolic profile biased toward glycolysis, which further influences CD4^+^ T cell cytokine production and their capacity to worsen NAFLD. Changes in the metabolic processes of effector T cells can also affect the occurrence and progression of liver cancer. For instance, miR-21 promotes CD4^+^ T cell glycolysis through the PTEN/PI3K/AKT pathway, which increases the cell cycle, promotes CD4^+^ T cell polarization toward the Th2 phenotype, and releases the fibrogenic cytokine IL-13, which is involved in arsenite-induced hepatic fibrosis [110] (Fig. 5A). The role of miR-21 in arsenic-induced fibrosis remains unclear. A conditional deletion of miR-21 in animal models is necessary to confirm its involvement in this process. In AIH models, glucose metabolism stimulates CD4^+^ T cell activation and concomitant inflammatory liver damage [111]. Various types of T cells play distinct roles in developing liver cancer. T cell function in the TME is affected by lactate, lactic acid inhibits the effector functions of CD4^+^ and CD8^+^ T cells. However, lactate promotes Treg stability and function. Tregs express more PD-1 than effector T cells in highly glycolytic malignancies, such as MYC-amplified tumors and liver cancers. Lactate can stimulate the growth of liver cancer stem cells and contribute to the progression of liver cancer. Elevated lactate levels promote Treg cell proliferation and enhance OXPHOS functions [112]. However, lactic acid inhibits the effector functions of CD4^+^ and CD8^+^ T cells. Tregs actively absorb lactic acid via monocarboxylate transporter 1 (MCT1) in low-glucose environments, which promotes NFAT1 translocation into the nucleus, thereby increasing liver tumors PD-1 expression [50]. Inhibition of MCT4 expression in HCC cells reduces tumor growth in immunocompetent mice by enhancing CD8^+^ T cell infiltration and cytotoxic activity [113]. Furthermore, Glycolysis is solely responsible for enhancing the production of IFN-γ by CD8 TILs under glucose-rich conditions. During low-glucose conditions, fatty acid oxidation, autophagy-dependent glutaminolysis, or both have been implicated [114]. Overall, the competition for glucose metabolism between T cells and liver cancer cells influences the onset and progression of liver cancer. The function of effector T cells and the immunosuppressive function of Treg cells can be enhanced by inhibiting the glycolysis of liver cancer cells. Further studies of the molecular pathways and interactions are needed.

### The role of amino acids metabolism between T cells and liver cancer cells

Amino acid metabolism plays an important role in HCC [115]. Tumor cells can suppress T cell immunity by reducing amino acids in the microenvironment because of nutritional competition, whereas toxic amino acid metabolites can impair T cell function. Furthermore, amino acids may compete with T cells by regulating glucose and lipid metabolism (Fig. 5B).

Tumor cells compete with T cells for amino acids to induce rapid proliferation and metastasis, while T cells attempt to mount an efficient antitumor response in the TME. Liver cancer cells often express certain amino acid transporters at high levels, enabling them to compete effectively with T cells for amino acids. For instance, Tumor cells outcompete T cells for cystine absorption due to their high Slc7a11 expression. This rivalry causes T cell exhaustion and ferroptosis, decreased memory formation and cytokine release, increased PD-1 and TIM-3 expression, and intracellular oxidative stress. Furthermore, depletion of cysteine uptake in T lymphocytes reduces glutathione production and increases lipid peroxide accumulation [71]. The reduced uptake of amino acids by T cells contributes to disease remission during hepatitis. For example, blocking amino acid metabolism in T cells can decrease the incidence of AIH. Inhibiting glutamine metabolism reduces T cell activation and the differentiation of Th1/Th17 cells by decreasing the mRNA expression of SLC7A5 and blocking the mTOR signaling pathway in AIH [116]. IDO1 is a Kyn-producing enzyme that affects tumor immune escape. Upregulation of IDO1 expression in HCC cells converts tryptophan I to Kyn, suppressing T cell activity. Furthermore, CD8^+^ T cells use IDO1 by the Kyn pathway to produce nicotinamide adenine dinucleotide (NAD), which is essential for metabolism and antitumor activity [117]. IDO1 inhibitors increase the number of CD4^+^ or CD8^+^ T cells, decrease Tregs, and block the expression of IDO1, CD47, and PD-L1, which inhibits HCC growth [118]. Tumor ethionine metabolic activity is associated with the degree of T cell dysfunction in tumor-associated liver tissues.T cell exhaustion in HCC patients is strongly linked to S-adenosylmethionine (SAM)/5-methylthioadenosine (MTA) levels in the TME [119]. Overexpression of MAT1A increases SAM levels, which induces ferroptosis of HCC cells through increased CD8^+^ T cell cytotoxicity and IFN-γ expression [120].

Glutamine metabolism is important for cell proliferation and the activation of CD8^+^ T lymphocytes which destroy cancer cells. Depriving HCC cells and CD8^+^ T cells of glutamine simultaneously enriches tumor-infiltrating CD8^+^ T cells and increases the efficacy of immunotherapy. In the glutamine-dominant HCC subgroup, CD8^+^ Tef cells shift to metabolizing exogenous lipids resulting from limited access to glutamine, reducing their cytolytic function [121]. HCC cells compete for excess glutamine, which leads to a decrease in T cell function. In addition, glutamine deprivation inhibits the activity of invading CD8^+^ T lymphocytes in HCC through mitochondrial damage and apoptotic pathways [122]. Spermine levels are elevated in HCC tumor tissues, and it plays a crucial role in the proliferation of HCC cells. Additionally, it can inhibit the proliferation and activation of CD4^+^ T cells in vitro, which promotes liver cancer. Spermine promotes liver tumor growth by activating the calcium-sensing receptor (CaSR), leading to increased PD-L1 expression and decreased CD8^+^ T cell infiltration. This occurs through the stabilization and movement of β-catenin to the cell nucleus, activating the expression of PD-L1 and STT3A, increasing PD-L1 stability in liver cancer cells [123, 124].

### The role of lipids metabolism between T cells and liver cancer cells

Lipid metabolism has been associated with the growth of HCC in patients with NAFLD (Fig. 5C). Lipid accumulation is a key characteristic of NASH and NAFLD, and excessive cholesterol uptake by CD8^+^ T cells can impair their effector function. Lipid accumulation in liver cells increases the risk of carcinogenesis. For instance, the reduced fatty acid metabolism in the liver leads to an accumulation of lipids in the TME and an increased uptake of lipids by CD8^+^ T cells. This process decreases the mitochondrial membrane potential of CD8^+^ T cells and contributes to their exhaustion, ultimately promoting the development of MASH-HCC [96]. HCC cells produce large amounts of cholesterol in the TME by expressing SREBP2 and its cholesterol synthesis gene. However, unlike glucose and part of amino acids, excessive cholesterol uptake by CD8^+^ T and CD4^+^ T cells inhibits their function. Cholesterol overuptake inhibited the secretion of GZMB and IFN-γ by CD8^+^ T and CD4^+^ T cells, promoting PD-1 expression in these T cells [125, 126]. The accumulation of cholesterol leads to fatty degeneration of liver cells, which is more likely to progress to HCC. Furthermore, Excessive cholesterol synthesis by hepatocytes results in lipid peroxide buildup and reduced cytotoxicity in NKT cells. Obesity-induced hepatic cholesterol buildup suppresses NKT cell-mediated antitumor immunity [127]. Similarly, in NAFLD-associated HCC, increased cholesterol and cholesteryl ester production inhibits CD8^+^ T cell activity in the TME. METTL3 promotes the translation of SREBP cleavage-activating protein (SCAP) mRNA m6A, which activates cholesterol production. This increases the release of cholesterol and cholesteryl esters and impairs CD8^+^ T cell activity in the TME [128]. METTL3 is a promising target for treating NASH; however, the study does not investigate the function and role of METTL3 in NAFLD. Targeting the cholesterol metabolism of tumors through sterol O-acyltransferase 1 (SOAT1)-targeted drugs alters tumor cholesterol metabolism and increases CD8^+^ T lymphocytes and neutrophils, thus preventing HCC development [129]. While only several SOAT1 inhibitors—avasimibe, nevanimibe, and CI-976- are effective, this severely limits the potential of medication research.

Intrahepatic CD4^+^ T cells are important for antitumor surveillance in NAFLD. Lipid metabolism in the fatty liver affects the onset and progression of fatty liver disease via CD4^+^ T cells. In NAFLD, disruption of lipid metabolism results in a selective decline in intrahepatic CD4^+^ T cells, but not CD8^+^ T cells, accelerating hepatocarcinogenesis. Linoleic acid supplies energy to T cells in HCC within the TME. However, an excessive accumulation of linoleic acid in NAFLD leads to a selective decrease in CD4^+^ T cells, which contributes to the progression of NAFLD to liver cancer. HCC cells compete for more long-chain fatty acids (LCFAs), such as linoleic acid, by overexpressing LCFA transporters. This competition leads to T cell dysfunction and promotes liver cancer progression [19]. In contrast, due to excessive accumulation of linoleic acid in NAFLD. Linoleic acid inhibits the antitumor ability of CD4^+^ T cells in the NAFLD. It disrupts mitochondrial activity and causes increased oxidative damage compared with other free FAs, such as palmitic acid. This results in the selective reduction of intrahepatic CD4^+^ T cells [130]. Ma et al. reported that CD4^+^ T cells generate more mitochondrial-derived ROS than CD8^+^ T cells. Hepatic lipid buildup is observed in HFD mice, with elevated linoleic acid, decreased apoptosis, and decreased CD4^+^ T cells [131]. An in-depth study is required to understand the role of linoleic acid in NAFLD and its molecular mechanism in NAFLD-related HCC. The carnitine palmitoyltransferase (CPT) family controls mitochondrial β-oxidation. The upregulation of the CPT gene increases mitochondrial ROS and results in the death of CD4^+^ T cells. Blocking CPT reduces the apoptosis of CD4^+^ T cells in the liver and prevents the development of NAFLD-related HCC [132]. Further studies are needed to understand how hepatocytes and other immune cells respond to CPT inhibition and their contribution to the development of HCC. HCC tissues show an increase in long-chain acylcarnitines (LCAC) and exhibit abnormal lipid metabolism. LCAC accumulates, leading to T cell depletion and deficiency. Exogenous LCAC inhibits NKT cell growth and promotes senescence. In contrast, lysophosphatidylcholines (LPCs) are associated with T cell antitumor effects. LPCs activate and secrete IFN-γ in T cells in vitro. LPCs can inhibit the growth of HCC in vivo [133, 134]. Further investigation is needed to determine whether inhibiting LCAC accumulation can prevent or delay cellular senescence. These data indicate that altering lipid metabolism may be used to prevent and treat HCC in HBV and NAFLD patients.

### The role of Mitochondria metabolism between T cells and liver cancer cells

T cell activity is influenced by mitochondrial quality and abundance. Inhibiting mitochondrial function in HCC cells lowers their growth. Similarly, blocking mitochondrial function in CD8^+^ T cells impairs effector function. The disruption of mitochondrial function directly impacts the antitumor activity of CD8^+^ T cells, thus promoting tumor formation and progression. Chronic infections and malignancies subject T cells to continual antigen stimulation and inflammatory signals. Mitochondrial dysfunction is a hallmark of deterioration during T cell exhaustion (Fig. 5D). The mitochondrial membrane potential (MMP) in CD8^+^ T cells from patients with HBV-HCC is significantly lower during the transition from chronic HBV infection to cirrhosis. Additionally, there was a negative link between the MMP of CD8^+^ T cells and AST, but the mitochondrial mass of CD8^+^ T cells correlated positively with AST. The unique metabolic characteristic of CD8^+^ T cells can be a diagnostic tool for assessing the severity of HBV-HCC disease [135]. CD4^+^ T cells are crucial in treating HCC associated with the hepatitis C virus (HCV). HCV infection in healthy CD4^+^ T cells blocks Top1 protein production and enzymatic activity, which results in mitochondrial dysfunction, topological mtDNA damage, and programmed cell death through numerous signaling pathways. Top1mt protein therapy may improve mtDNA structure and T cell function in patients with chronic HCV infections [136]. Hepatic mitoribosome abnormalities enhance glucose partitioning towards glycolytic flux and lactate production, which leads to T cell exhaustion and cancer progression. Hepatocyte-specific mitochondrial ribosomal protein CR6-interacting factor 1 (CRIF1) deficiency increases PD-1 expression and creates an adverse environment for T cells, which results in T cell fatigue due to elevated reactive oxygen species and lactate generation [137]. Mtfp1 deletion prevents mitochondrial permeability transition pore opening in hepatocytes, protecting against apoptotic liver damage both in vivo and ex vivo [138]. Furthermore, CRIF1 plays an important role in the progression of HCC in vivo. Song et al. demonstrated mitochondrial ribosomal protein CRIF1 deficiency leads to a negative environment for T lymphocytes, which results in T cell exhaustion due to elevated reactive oxygen species and lactate production in liver cancer progression [137]. miR-23a targets PPIF, the gatekeeper of the mitochondrial permeability transition pore, reduces ROS flux and maintains mitochondrial integrity. Deleting miR-23a in T cells causes significant inflammation and severe liver damage [139]. Therefore, improving mitochondrial metabolism in T cells is a rational approach for inducing anti-HCC immunity.

### Targeting T cell metabolism in the treatment of liver cancer

Competition between tumor cells and TILs for glucose uptake can lead to glucose deficiency in the TME and lactate accumulation. This metabolic imbalance inhibits the function of CD8^+^ TILs and supports the immunosuppressive activity of Tregs. However, suppressing aerobic glycolysis and destabilizing the mitochondria in cancer cells can destabilize Tregs and enhance the activation and killing activity of CD8^+^ T cells [140] (Fig. 5).

Generally, the high infiltration of CD8^+^ T cells predicts improved clinical outcomes for HCC patients. The increased infiltration of Treg cells negatively impacts liver cancer prognosis. HBV infection patients exhibiting Treg cell accumulation in the liver have a high risk of HCC development [141]. Current microwave therapy can inhibit liver cancer. It has already been implemented in clinical practice. MWA alone shows a significant antitumor effect in the immunologically “hot” tumor models, but not in the immunologically “cold” tumor models. Current literature also suggests that glycolysis inhibitor combination with MWA therapy is an effective method for boosting T cell killing ability and inhibiting the growth of liver cancer. Peripheral CD8^+^ T cells from patients with liver cancer treated with microwave ablation (MWA) and glycolysis inhibition are differentiated into central memory CD8^+^ T cells (TCM) cells. This combination leads to a long-term antitumor effect by enhancing the differentiation of tumor-specific CD8^+^ TCM cells. Additionally, the combination of microwave therapy with molecular targeted therapy has proven to be an effective approach for treating liver cancer. Combining MWA and Gr-1 inhibiting therapy can greatly improve CD8^+^ T cells and limit HCC growth. Some studies have shown that targeting PGAM1 in HCC cells may transform “cold” tumors into “hot” tumors with an inflammatory TME [105, 142, 143]. The combination therapy of MWA and targeting PGAM1 presents a promising research direction for the treatment of liver cancer. Abrine can inhibit amino acid metabolism in HCC cells and enhance T cell amino acid metabolism competition. The combination therapy of Abrine (IDO1 inhibitors) with anti-PD-1 antibody has a beneficial impact on decreasing HCC growth by upregulating CD4^+^ or CD8^+^ T cells, inhibiting Treg cells, and blocking the expression of IDO1 and PD-L1. Abrine may inhibit the expression of PD-L1 in tumor tissues [118]. New therapeutic targets for intracellular cholesterol homeostasis are expected to accelerate the development of liver cancer therapies. The SOAT1 protein converts excess intracellular cholesterol into cholesterol ester, which is then stored in lipid droplets. By controlling cholesterol homeostasis in tumor cells, inhibiting or knocking down SOAT1 can effectively suppress various types of cancer. In HCC, drugs such as Nilotinib and ABT-737 which target SOAT1, have been found to alter cholesterol metabolism and enhance CD8^+^ T cells and neutrophils killing ability, thus inhibiting HCC growth [129, 144, 145]. Traditional Chinese medicine (TCM) therapy also has a perfect curative effect. For instance, The water-soluble polar components of the Dahuang Zhechong pill (DHZCP) inhibit Treg differentiation and enhance the tumor-killing ability of CD8^+^ T cells by reducing hepatoma cell metabolism, lowering TME acidity, and depleting glutamine. DHZCP has multi-targeted synergy action, whereas TCM has many components and multitarget action, linking a single ingredient and a pharmacological effect [146]. A new study is needed to understand the link between changes in pharmaceutical composition and pharmacological effects. Metformin can suppress non-diabetic HCC by increasing all-trans-retinoic acid (atRA) levels while decreasing CD8^+^ T cells [147]. Metformin is an effective treatment for the prevention and management of liver cancer. Additionally, metformin can enhance T cell function. Metformin promotes IFN-γ, TNF-α production, and CD25 expression in CD8^+^ T cells after TCR stimulation [114]. Metformin can improve the prognosis of patients with NASH. It can also inhibit the proliferation of HCC cells. Furthermore, metformin suppresses the downregulation of genes related to various metabolic pathways in CD8^+^ T cells in NASH, which improves mitochondrial activity [109]. Understanding the benefits and drawbacks of inhibitors and leveraging their therapeutic potential in clinical applications remains a significant challenge. Molecules that target T cell metabolism hold significant research potential for improving diseases related to liver cancer, such as liver fibrosis. For example, miR-21 modulates glycolysis in CD4^+^ T cells through the PTEN/PI3K/AKT pathway, accelerates the cell cycle, promotes CD4^+^ T cell polarization toward Th2, and induces fibrogenic cytokine IL-13 expression, which participates in arsenite-mediated fibrosis of the liver [110]. In summary, understanding the metabolic pathways that regulate immune cell activity in the TME is important for the development of effective cancer therapeutics. These results suggest that therapeutic interventions targeting metabolic pathways in T cells have the potential to enhance the antitumor immune response and improve treatment outcomes.

## Conclusions

T cells are important for combating infections and malignancies, and T cell-based immunology is emerging as a potential cancer treatment. Studies have focused on determining how the metabolism of liver cancer cells affects T cell metabolism, which in turn, modulates T cell-mediated antitumor immunity. However, there are few studies on the molecular mechanism of T cell metabolism. In NASH, the metabolism of T cells is inhibited; however, the exact mechanism of action remains unclear. Future studies should investigate whether T cells can improve the inflammatory response of NASH through metabolism. Furthermore, targeting the metabolism of liver cancer cells can potentially improve T cell antitumor immunity. When T cells are activated, the metabolism of T cells shifts from oxidative phosphorylation to glycolysis, providing energy for rapid growth and enhanced function. Increasing the glucose metabolism capacity of T cells can improve their tumor infiltration and killing ability in liver cancer. Similarly, decreasing glycolysis in the liver cancer cells can boost the metabolism of T cells and enhance their function.

In the TME, activated T cells and tumor cells compete for energy sources, such as glucose, amino acids, and lipids; however, glucose deficiency, lactate buildup, and lipid uptake can influence T cell activation and energy metabolism. Regulating T cell metabolism can improve liver cancer sensitivity to immunotherapy and chemotherapy. These findings emphasize the role of target T cell metabolism in liver cancer progression. Further study is needed to clarify the detailed mechanisms. Modulating glucose, amino acid, and lipid metabolism in T cells enhances their cytotoxicity. More clinical data are required to fully understand target T cell metabolism’s therapeutic potential for liver cancer. In addition, future clinical trials should investigate if targeting T cell metabolism is more effective in certain liver cancer patient types. Combining immune checkpoint and T cell metabolism targeting therapy shows promise for treating HCC. Despite limited studies in recent years, enhancing T cell metabolism holds significant promise for treating liver cancer, particularly when combined with immune checkpoint inhibitors.
